# Co-Graft of Allogeneic Immune Regulatory Neural Stem Cells (NPC) and Pancreatic Islets Mediates Tolerance, while Inducing NPC-Derived Tumors in Mice

**DOI:** 10.1371/journal.pone.0010357

**Published:** 2010-04-27

**Authors:** Raffaella Melzi, Barbara Antonioli, Alessia Mercalli, Manuela Battaglia, Andrea Valle, Stefano Pluchino, Rossella Galli, Valeria Sordi, Emanuele Bosi, Gianvito Martino, Ezio Bonifacio, Claudio Doglioni, Lorenzo Piemonti

**Affiliations:** 1 San Raffaele Diabetes Research Institute (HSR-DRI), Division of Immunology, Transplantation and Infectious Disease, San Raffaele Scientific Institute, Milan, Italy; 2 CNS Repair Unit, Institute of Experimental Neurology (INSPE), Division of Neuroscience, San Raffaele Scientific Institute, Milan, Italy; 3 Neural Stem Cell Biology Unit, Division of Regenerative Medicine Stem Cells and Gene Therapy, San Raffaele Scientific Institute, Milan, Italy; 4 Diabetes and Endocrinology Unit, Department of Internal Medicine, San Raffaele Scientific Institute, Milan, Italy; 5 Neuroimmunology Unit, Institute of Experimental Neurology (INSPE), Division of Neuroscience, San Raffaele Scientific Institute, Milan, Italy; 6 Center for Regenerative Therapies Dresden, Dresden University of Technology, Dresden, Germany; 7 Pathology Unit, San Raffaele Scientific Institute and Università Vita–Salute, Milan, Italy; New York University, United States of America

## Abstract

**Background:**

Data available on the immunomodulatory properties of neural stem/precursor cells (NPC) support their possible use as modulators for immune-mediated process. The aim of this study was to define whether NPC administered in combination with pancreatic islets prevents rejection in a fully mismatched allograft model.

**Methodology/Principal Finding:**

Diabetic Balb/c mice were co-transplanted under the kidney capsule with pancreatic islets and GFP^+^ NPC from fully mismatched C57BL/6 mice. The following 4 groups of recipients were used: mice receiving islets alone; mice receiving islets alone and treated with standard immunosuppression (IL-2Rα chain mAbs + FK506 + Rapamycin); mice receiving a mixed islet/NPC graft under the same kidney capsule (Co-NPC-Tx); mice receiving the islet graft under the left kidney capsule and the NPC graft under the right kidney capsule (NPC-Tx). Our results demonstrate that only the co-transplantation and co-localization of NPC and islets (Co-NPC-Tx) induce stable long-term graft function in the absence of immunosuppression. This condition is associated with an expansion of CD4^+^CD25^+^FoxP3^+^ T regulatory cells in the spleen. Unfortunately, stable graft function was accompanied by constant and reproducible development of NPC-derived cancer mainly sustained by insulin secretion.

**Conclusion:**

These data demonstrate that the use of NPC in combination with islets prevents graft rejection in a fully mismatched model. However, the development of NPC-derived cancer raises serious doubts about the safety of using adult stem cells in combination with insulin-producing cells outside the original microenvironment.

## Introduction

Adult multipotent neural stem/precursor cells (NPC) are broadly proposed as an alternative cell source to repair brain damage upon transplantation and NPC-driven brain repair has variably been shown in several pre-clinical models of neurological disorders [1], [2]. While the replacement of lost or damaged cells was until a few years ago assumed to be the prime therapeutic mechanism of stem cell, it is now clear that transplanted NPC may simultaneously instruct several mechanisms not confined to cell replacement on its own [1]. In experimental autoimmune encephalomyelitis models, NPC exert immune-like functions and induce apoptosis of blood-borne encephalitogenic T cells [3]–[6], as well as decrease CNS inflammation via peripheral suppression of the adaptive immune response [6]–[8]. In an intracerebral haemorrhage model, NPC have an important “bystander” anti-inflammatory effect on the spleen-macrophage system [9]. Moreover *in vitro* NPC directly inhibit T-cell activation and proliferation [3], [10], [11]. These evidences support the concept that the “therapeutic plasticity” [1] and in particular the immunomodulatory activity is a true functional signature of NPC. Nonetheless, the recent demonstration that other sources of somatic stem cells (*i.e.* mesenchymal, haematopoietic), with very low capabilities of neural (trans) differentiation, may show equally significant bystander capacities and promote CNS repair [12]–[14], further proves and generalizes the relevance of somatic stem cell-dependent alternative therapeutic mechanisms.

Allogeneic islet transplantation serves as a source for insulin-secreting beta cells for the maintenance of normal glucose levels and treatment of diabetes [15]. However, limited availability of islets, high rates of the islet graft failure and the need for life-long non-specific immunosuppressive therapy have been major obstacles in the widespread adoption of this therapeutic approach [16]. Recently, pancreatic islet transplantation was suggested as a potential target of the “therapeutic plasticity” of adult stem cells. In rat, Solari et al demonstrated that mesenchymal stem cells promote long-term isl allograft survival in the presence of short-term immunosuppression [17]. In mouse, we demonstrated that tissue-derived adult mesenchymal stem cells, when co-transplanted with a minimal pancreatic islet mass, facilitate restoration of normoglycemia and neovascularization of the graft [18]. In humans, combined islet and hematopoietic stem cell allotransplantation was attempted to prevent graft rejection and avoid immunosuppression-related side effects [19].

The aim of this study was to define whether NPC administered in combination with islets prevents acute pancreatic islet allograft rejection. Our results demonstrate that in diabetic mice receiving allogeneic islet transplantation, the co-transplantation and co-localization of allogeneic NPC and islets induces stable long-term islet function that is not reversed by alloantigen re-challenge. This condition is associated with splenic expansion of T cells with a regulatory phenotype (*i.e.* CD4^+^CD25^+^FoxP3^+^). However, stable graft function was accompanied by constant and reproducible development of NPC-derived cancer which was sustained by insulin secretion. Our results demonstrate that the immune-like functions of NPC are efficient also in non-neurological disorders but raises serious doubts about the safety of using adult stem cells outside the original microenvironment in combination with insulin-producing cells.

## Results

### NPC co-transplantation and co-localization induce long-term graft tolerance in fully mismatched islet transplants

**Figure 1:**
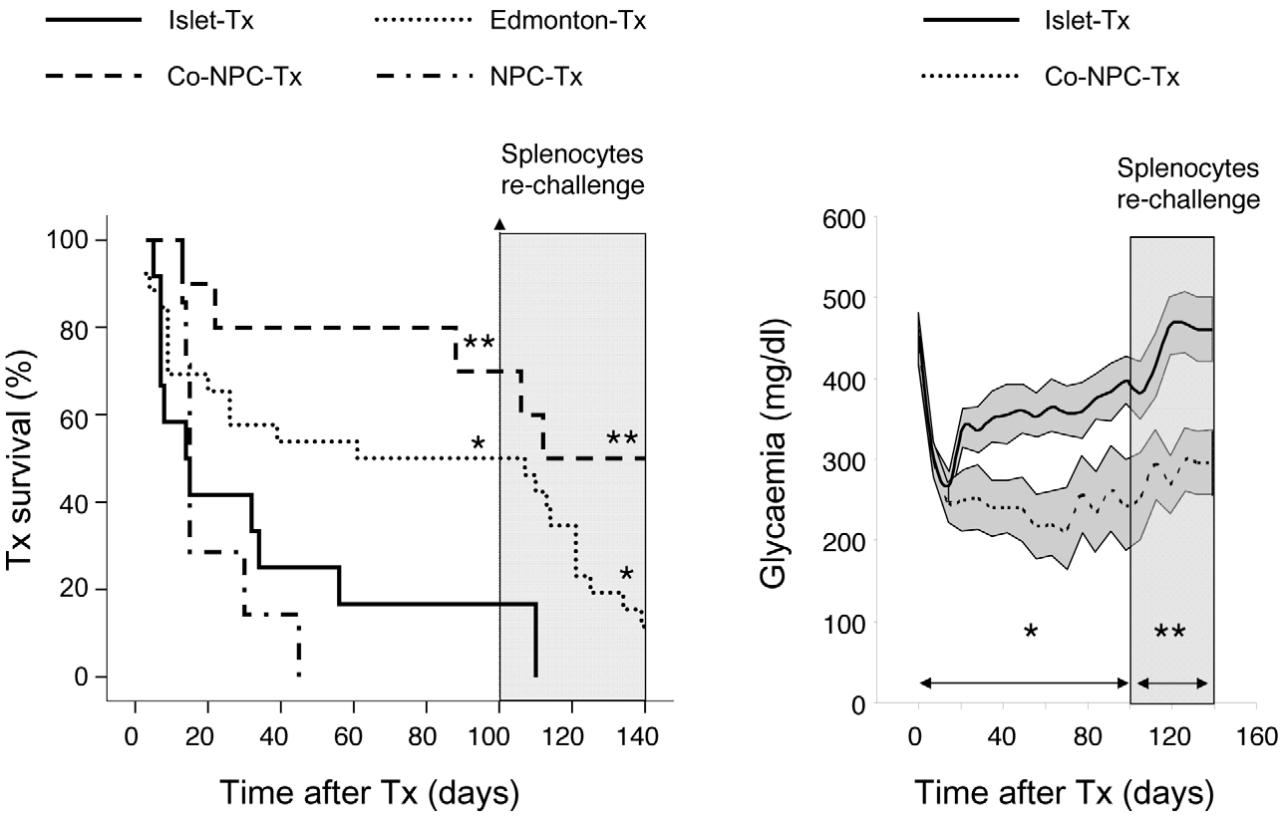
NPC/islet cotransplantation and colocalization prevents allograft rejection. Diabetic Balb/c mice were transplanted under the kidney capsule with 350 EI purified from C57BL/6 mice. Mice were not treated (Islet-Tx, *n* = 12) or treated with rapamycin plus FK506 plus anti–IL-2R*α* mAb (Edmonton-Tx, *n* = 26), or co-transplanted with NPC (1,000 neurospheres) under the same kidney of the islets (Co-NPC-Tx, *n* = 10) or under the controlateral one (NPC-Tx, *n* = 7). *Gray area:* all diabetic Balb/c mice that were transplanted 100 days before with C57BL/6 islets were challenged with donor-derived splenocytes. Left Panel: Kaplan-Meier Analysis for the graft survival between the four groups of recipients. Hundred days after transplantation there was a significant difference between Edmonton-Tx and Islet-Tx or between Co-NPC-Tx and Islet-Tx. In contrast, there was no statistical significant difference between NPC-Tx and Islet-Tx. In all cases, the graft survival was monitored by glycemia levels. A graft was considered rejected when glycemia was >300 mg/dl for two consecutive measurement. *P<0.05 and **P<0.01 vs. Islet-Tx, Log Rank-Kaplan-Meier analysis. Right Panel: not fasting blood glucose profile of Islet-Tx and Co-NPC-Tx after receiving islets. Data are expressed as mean (line) and ±1 standard error (area). ^a^ Statistical analysis was performed by tests of repeated measures ANOVA considering two intervals: from 0 to 100 days (before donor-derived splenocytes boost) and from day 100 to day 140 (after donor-derived splenocytes boost). * p<0.05; ** p<0.01.

Diabetic Balb/c mice were transplanted with pancreatic islets (350 EI) and/or NPC (1000 neurospheres, GFP^+^) from fully mismatched C57BL/6 mice. The following four groups of recipients were used: diabetic Balb/c mice receiving islets alone (Islet-Tx, n = 12); diabetic Balb/c mice receiving islets alone and treated with rapamycin plus FK506 plus anti–IL-2Rα chain mAbs (Edmonton-Tx, n = 26); diabetic Balb/c mice receiving a mixed islet/NPC graft under the left kidney capsule (Co-NPC-Tx; n = 10); diabetic Balb/c mice receiving the islet graft under the left kidney capsule and the NPC graft under the right kidney capsule (NPC-Tx; n = 7) (Figure 1).

In the Islet-Tx group, the allograft was lost at a median survival time (MST) of 15±3 days and 16.7% (2/12) of allografts survived without evidence of rejection for 100 days. The presence of immunosuppression (Edmonton-Tx) delayed graft rejection (MST 61±10 days; p = 0.05 vs Islet-Tx) and 50% (13/26) of allografts survived for 100 days (p = 0.05 vs Islet-Tx; Pearson Chi-Square test). NPC co-transplantation and co-localization with islets (Co-NPC-Tx) prevented graft rejection (MST >100 days; p = 0.007 vs Islet-Tx) and 70% (7/10) of allografts survived for 100 days (p = 0.01 vs Islet-Tx). In contrast, when NPC were co-transplanted but not co-localized with islets (NPC-Tx) the graft MST was 15±1 days (p = 0.5 vs Islet-Tx) and none of the allografts (0/7) survived up to 100 days (p = 0.2 vs Islet-Tx). To determine whether mice that had islet graft survival >100 days had an active state of tolerance, all transplanted mice were rechallenged *in vivo* with splenocytes isolated from donor mice [20]. After the injection of donor splenocytes, 100% of mice within the Islet-Tx group, 77% within the Edmonton-Tx group and only 28% within the Co-NPC-Tx group rejected their islet graft. These results indicate that no active tolerance was achieved in the mice within the Islet-Tx and Edmonton-Tx groups since the injection of donor-derived antigens broke the state of immune incompetence leading to graft rejection [20]. On the contrary, Co-NPC-Tx mice displayed a state of active and stable long-term tolerance that was not broken by alloantigen rechallenge. Overall, the proportion of mice achieving long-term tolerance (*i.e.* accepting the primary graft and retaining the graft after rechallenge) was 50% in the Co-NPC-Tx group, 13% in the Edmonton-Tx group and 0% in Islet-Tx and NPC-Tx groups (Table 1).

**Table 1:** Percentage of mice in which stable long-term graft survival was achieved after different treatments.

Transplant group	Kidney capsule		n	100 days after Tx		After rechallenge		Overall	
	Left	Right		GS	MST	GS	MST	GS	MST
Islet-Tx	Islet	Sham	12	2/12	15*±*3	0/2	7	0/12	14±6
Edmonton-Tx	Islet	Sham	26	13/26 *	61±10*	3/13	21*±*4	3/26	61±53§
Co-NPC-Tx	Islet/NPC	Sham	10	7/10 §	>100§	5/7	>40	5/10#	112#
NPC-Tx	Islet	NPC	7	0/7	15±1	-		0/7	15±1

Graft survival (GS): data are *n*. Median survival time (MST): data are days ±standard error.

*P≤0.05 vs Ctrl;

§ *P*≤0.01 vs Ctrl;

# *P*≤0.005 vs Islet Tx.

### Islet/NPC co-transplantation and co-localization induce NPC transformation and tumor formation

**Figure 2:**
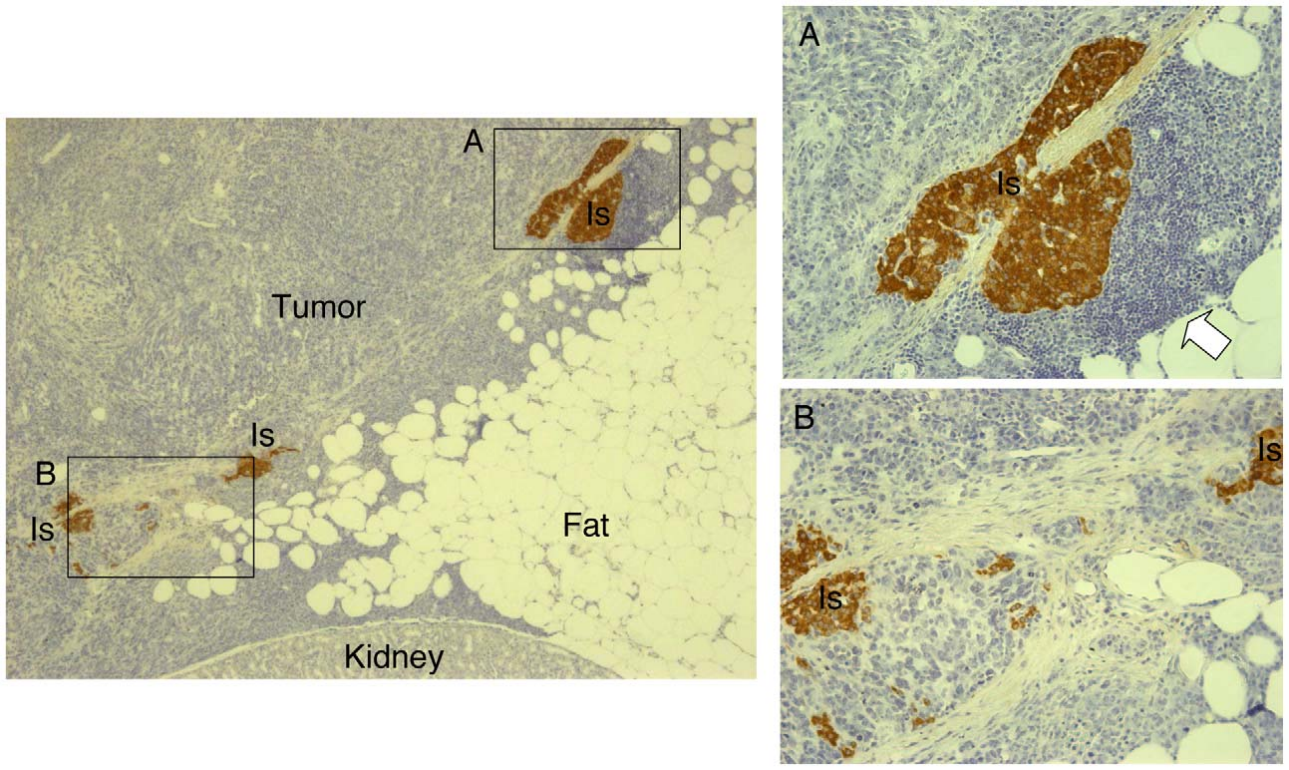
Co-NPC-Tx: transplant histology. Histological appearance (left 4x, insert 20x) of allogeneic islet/NPC co-transplantation in a representative Balb/c mouse 140 days after transplantation. Islets (Is) appeared embedded in tumor tissues, localized preferentially close to kidney tissue and stained strongly for insulin (*brown*). The frequency of leucocytes infiltrating the tumors and more specifically the islets, was very low (Insert B). However, when present, infiltrating cells were confined to the periphery of the islets whereas intra-islet infiltrating mononuclear cells were not observed (insert A, white arrow).

All mice that were still alive 140 days after allo-transplant (11/12, 6/7 and 10/10 for Islet-Tx, NPC-Tx and Co-NPC-Tx respectively) were sacrificed. Concordantly with the presence of hyperglycaemia, all mice within the Islet-Tx group (11/11), the NPC-Tx group (6/6), and the Co-NPC-Tx group (3/10) that had lost islet function during the first 100 day, had neither macroscopic nor microscopic residual grafted tissue (islet and/or NPC) under their kidney capsules. No GFP+ cells were present in the draining lymph nodes or in the spleens. On the other hand, all seven mice within the Co-NPC-Tx group that did not reject the graft 100 days after transplantation had tumors at the site of islet/NPC implantation (Table S1 and Figure S1). All tumors stained positive for GFP, showing that they derived from the grafted NPCs. In the five mice that maintained euglycaemia after alloantigen rechallenge, histological analysis demonstrated insulin positive islets embedded within the tumor and localized preferentially close to the kidney (Figure 2). The frequency of leucocytes infiltrating the tumors and more specifically the islets, was very low. However, when present, infiltrating cells were confined to the periphery of the islets whereas intra-islet infiltrating mononuclear cells were not observed (Figure 2).

### Islet/NPC co-transplantation and co-localization associates with a splenic expansion of FoxP3^+^ and CD4^+^CD25^+^FoxP3^+^ T regulatory cells

The frequency of the major hematopoietic populations was analyzed in spleens of mice within the Islet-Tx, NPC-Tx, and Co-NPC-Tx groups and of untreated age and gender matched Balb/c mice (Table 2). Transplantation changed *per se* the splenocyte composition leading to decreased CD8^+^ T cells, influencing the naïve/effector T-cell ratio (Table 2, CD45RBhigh expression on CD4^+^ and CD8^+^ T cells), and favoring the expression of MHCII and costimulatory molecules on CD19^+^B220^+^ B cells and CD11b^+^ myeloid cells. Interestingly, among the transplanted mice, those within the Co-NPC-Tx group showed a significant reduction of the percentage of total CD4^+^ T cells with a concomitant significant expansion of the percentage of FoxP3^+^ and CD4^+^CD25^+^FoxP3^+^ regulatory T cells. Moreover, a further increase of MHCII and CD86 expression was evident in CD19^+^B220^+^ B cells and CD11b^+^ myeloid cells.

**Table 2:** Phenotype of spleen derived cells.

	Untreated Balb/c Mice N = 5	Islet-Tx N = 11	NPC-Tx N = 6	Co-NPC-Tx N = 10	P
CD4^+^	31±2	24±3	27±6	15±8 a ^,^ b ^,^ c	<0.001
- CD25^+^	5±1	18±7 a	10±6	26±8 a ^,^ c	<0.001
- FoxP3^+^	15+2	18±4	16±3	23±2 a ^,^ b ^,^ c	<0.001
- FoxP3^+^/CD25^+^	9+1	11±5	7±3	16±3 a ^,^ b ^,^ c	<0.001
- CD62L^+^	72±3	60±9	Nt	58±16	Ns
- CD25^+^/CD62L^+^	4±1	9±4 a	Nt	10±2 a	0.003
- CD45Rb high^+^	80±2	56±7 a	57±5 a	53±9 a	<0.001
CD8^+^	11±8	8±3 a	6±2 a	6±2 a	0.001
- CD45Rb high^+^	93±1	75±6^a^	71±6^a^	63±20^a^	0.002
CD14^+^	1±0.2	4±3	1±1	4±3	Ns
Gr1^+^	1±0.2	3±4	5±7	3±2	Ns
Gr1^low^/B220^+^	0.2±0.5	4±2 a ^,^ c	2±1	4±2 a ^,^ c	<0.001
CD19^+^/B220^+^	41±3	53±7	42±2	50±20	Ns
- I-Ab^+^	1.5±0.3	12±7 a	3±0.8	22±10 a ^,^ b ^,^ c	<0.001
- CD86^+^	0.5±0.5	21±14 a ^,^ c	7±3	37±7 a ^,^ b ^,^ c	<0.001
CD11b^+^	1.5±0.1	4.2±2.3	7.8±2.9	4.8±2.1 a ^,^ b ^,^ c	<0.001
- I-Ab^+^	1.14±0.6	67±37 a	33±25	89±8 a ^,^ c	<0.001
- CD80^+^	9±2	75±31 a	54±31 a	94±4 a ^,^ c	<0.001
- CD86^+^	9±2	73±32 a	46±30	89±14 a ^,^ c	<0.001

Data are expressed as percentage ±SD. Statistical analysis was performed by one-way ANOVA with Bonferroni post-hoc test:

a =  p<0.05 vs Balb/c Mice;

b =  p<0.05 vs Islet-Tx;

c =  p<0.05 vs NPC-Tx; ^d^  =  p<0.05 vs Co-NPC-Tx. Ns = not statistically significant; Nt =  not tested.

### Islets and insulin are required for NPC transformation and tumor formation

To evaluate whether the co-localization with islets is the *“sine qua non”* condition for promoting NPC transformation, 8 diabetic C57BL/6 mice were transplanted with syngeneic pancreatic islets (350EI) and syngeneic GFP^+^ NPC (1,000 neurospheres) under the left kidney capsule and with syngeneic GFP^+^ NPC alone (1,000 neurospheres) under the right kidney capsule (see Table S1). All mice normalized glycaemia. Islet function was maintained for 100 days after transplant with the exception of two mice: one died at day 85 and one lost islet function at day 78. At day 100, a tumor was present only in the left kidney in all seven surviving mice. Subsequently, to test whether tumor development was sustained by insulin secretion we substituted islets with an insulin releasing pellet. Three diabetic C57BL/6 mice were transplanted with syngeneic NPC (1,000 neurospheres) and sustained release insulin implants (release rate: ∼0.1 U/24 hr/implant for >30 days) under the left kidney capsule and with syngeneic NPC alone (1,000 neurospheres) under the right kidney capsule. After 140 days all animals (3/3) showed tumor formation in the left kidney (Figure S2). In the right kidney, at the level of NPCs implantation, fluid-filled microcysts were recognizable. These cysts were lined by a multiple layer of phenotypically non transformed neuroepithelial cells (Figure S2). All together these data demonstrate that insulin is a determinant of NPC transformation *in vivo*.

### NPC-derived tumors are neuroblastoma-like neoplasms with malignant features

**Figure 3:**
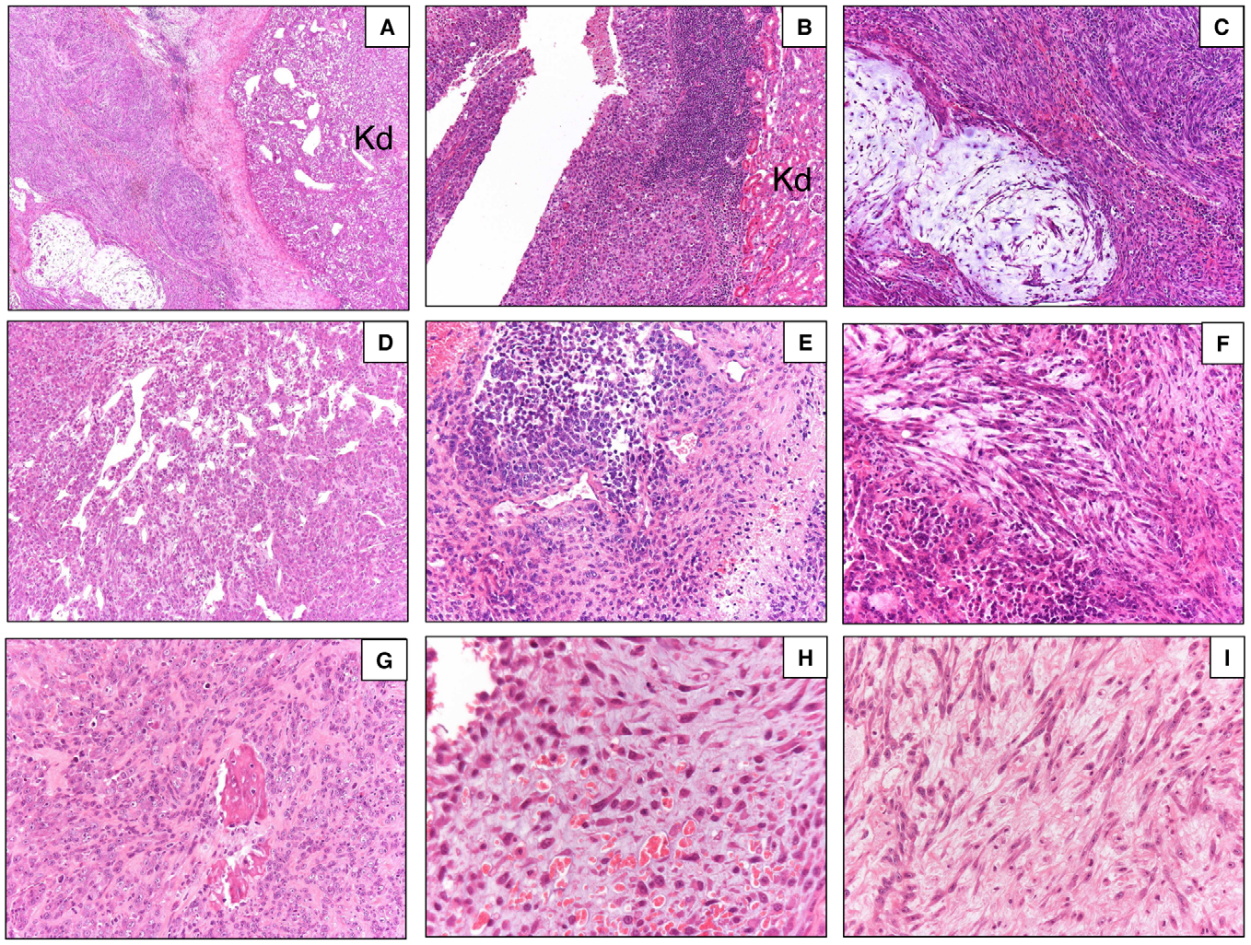
Co-NPC-Tx: tumor histology. Variable morphological appearances within the haematoxylin and eosin stained tumours: (A) neural and spindle mixoid components (4x); (B) neural component (10x); (C) spindle mixoid components(10x); (D) angiomatoid component (10x); (E) differentiated and undifferentiated neural components (20x); (F) neural and spindle component (20x); (G) osteoid and epitheliod components (20x); (H) condroid component (40x); (I) spindle component (40x). Kd = kidney.

The median volume of developed tumors was 3.1 cm^3^ (range 0.1–6.4). In 2 of 7 mice (28%) peritoneal and liver GFP+ metastases were present and associated with ascites. Tumors had a variable morphological appearance by histological evaluation (Figure 3). They generally appeared non-encapsulated, densely cellular with variable areas of necrosis (5 to 30%), well circumscribed although focally infiltrating adjacent tissues (Figure S3). Evaluation of Ki-67 staining showed an impressive high proliferation index in all tumors (positive cell >80%) (Figure S3). Features of differentiated and undifferentiated neuroblastoma were easily recognizable in large tumor areas (∼60%) with neuroepithelial cells (small cells S100^+^ with hyperchromatic nuclei and scant cytoplasm) sometimes arranged in the classic *pseudorosette* pattern (Figure 3). In addition to the neuroblastoma areas, spindle cell sarcomatoid components mainly as densely cellular areas and less frequently immersed in a myxoid stroma, were present. The spindle cell component stained positively, at least focally, for smooth muscle actin (data not shown). Moreover, within the tumors, it was possible to recognize foci of angiomatoid, epithelioid, condroid and osteoid differentiation (Figure 3).

**Figure 4:**
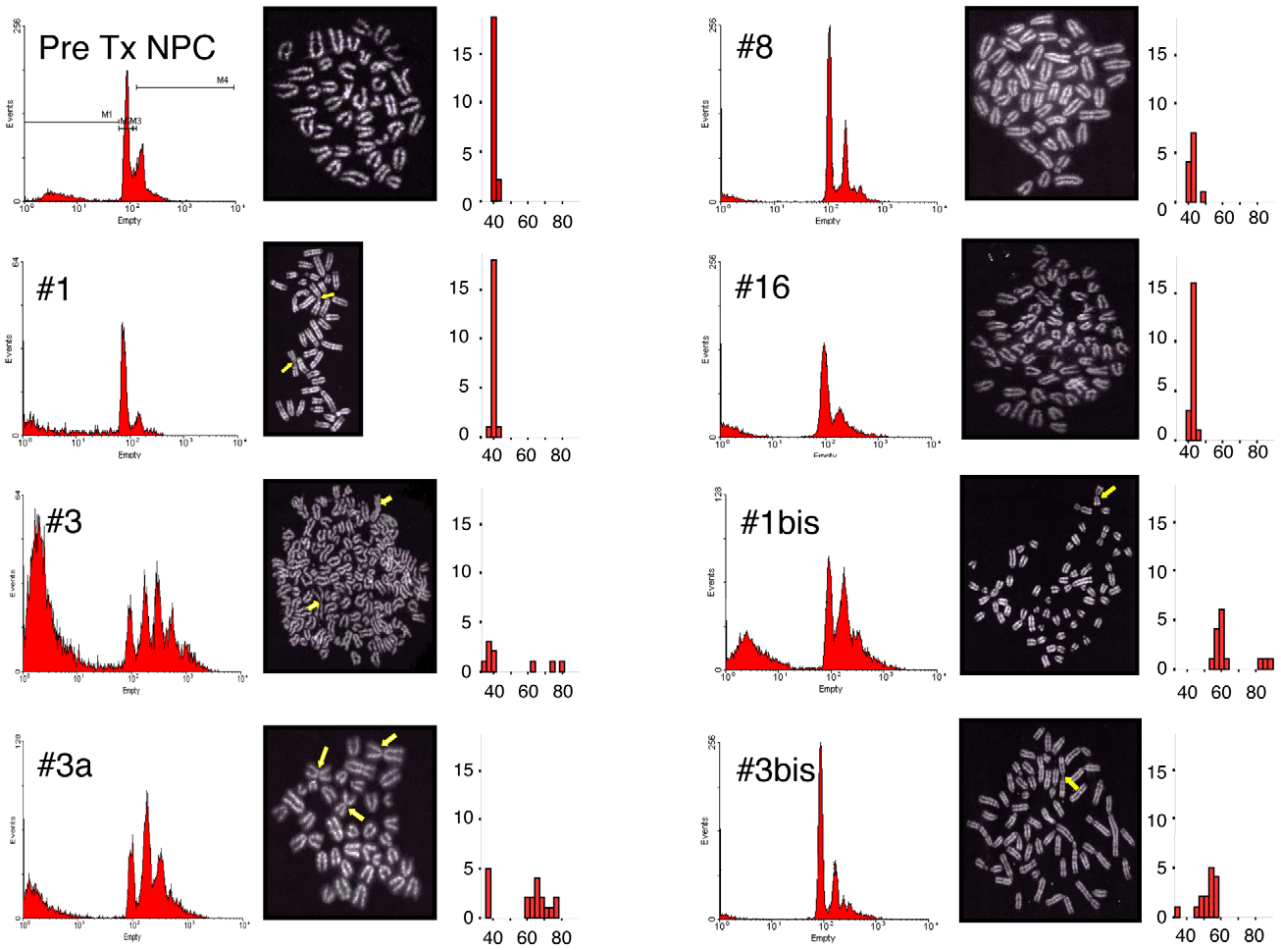
Cell-cycle and karyotype assessments of NPC-derived tumor cell cultures (NPC-DTCC). Cell-cycle profile, examples of metaphase and chromosome number distribution from karyotype analysis in pre Tx NPC (p15) and NPC-DTCC (#1, p8; #3, p3; #3a, p4; #8, p2; #16, p4, #1bis, p5; #3bis, p7). Sub G1, G1, S, G2 and hyper G2 fractions were calculated as reported by the markers in pre Tx NPC panel. Yellow arrows in metaphase picture indicate abnormal metacentric chromosomes. Chromosome number distribution: X axis represents the number of chromosome counted during the metaphase, Y axis represent the number of metaphase. A minimum of 9, a maximum of 20 metaphases were analyzed for each cell culture.

**Figure 5:**
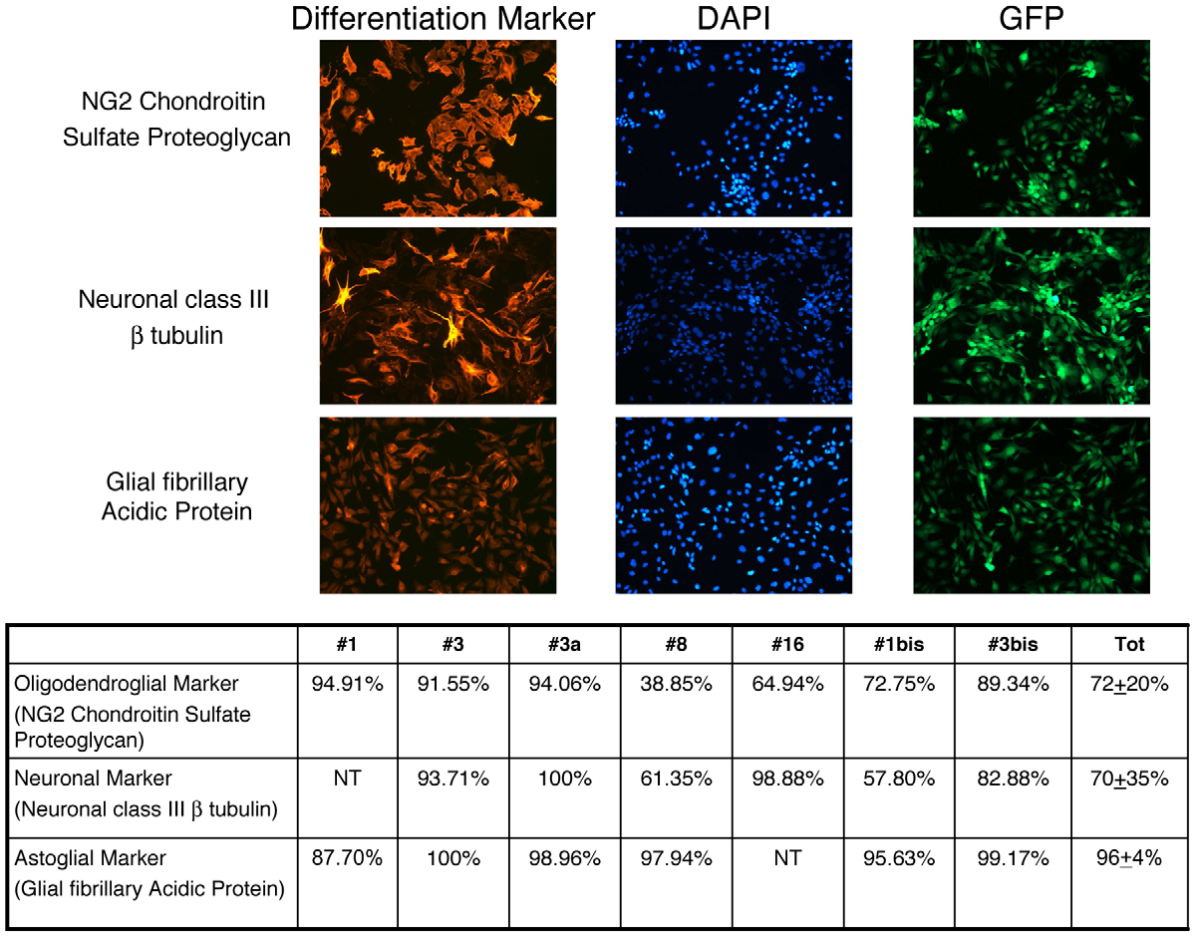
Multipotency of NPC-derived tumor cell cultures (NPC-DTCC). *Upper Panel*: Analysis of NPC-DTCC multipotency at passages 24 by immunofluorescence for neuronal (Neuronal class III beta tubulin), astroglial (Glial fibrillary Acidic Protein), and oligodendroglial (NG2 Chondroitin Sulfate Proteoglycan) markers (line #1, representative of all NPC-DTCC). All the cells in culture maintained the positivity for GFP, demonstrating their NPC origin *Lower Panel*: quantitative analysis of the frequency of CNS lineages in the NPC-DTCC at passages 16. Neuronal, glial, oligodendroglial markers were detected by immunofluorescence. Values are expressed as percentage of immunoreactive cells over the total cell number. A minimum of 100 and a maximum of 1500 cells were evaluated for each marker.

Primary adherent cell cultures were established from 4 of 14 primary tumors (#1, #3, #8, and #16) and from 1 of 2 ascites (#3a). All these NPC-derived tumor cell cultures (NPC-DTCC) generated “secondary” tumors *in vivo* when injected into syngeneic recipients. Two additional NPC-DTCC were established from these “secondary” tumors (#1bis, #3bis). All the cells in culture were and remained positive for GFP, demonstrating their original derivation from NPC (Figure S4). All NPC-TDCC maintained stable growth capacity *in vitro* (Figure S5 and Table 3) and continuously proliferated in serum-supplemented medium without any addition of growth factors for up to 32 passages (corresponding to 280 days). Cell cycle and Q-banding karyotypic analysis at early passages (passage 2–8) showed that aneuploidy and chromosomal aberrations frequently occur in NPC-TDCC (Figure 4). Less than 10% of total metaphases analyzed displayed euploidia and cell cycle analysis showed a significant increase of the hyper G2 fraction in comparison to pre-transplant NPC (Table 3). Moreover, we detected the presence of atypical metacentric chromosomes, probably as a result of chromosomal end-to-end fusions of Robertsonian type (Figure 4). The average cell number yielded per passage during *in vitro* culture ranged from 3.5×10^6^ at p1–p5 to 7.5×10^6^ at p6-p10 to 10^7^ at later passages (p11–16), suggesting that culturing resulted in an increased proliferation rate (Figure S5). The quantitative analysis of the frequency of CNS lineages in the NPC-DTCC (Figure 5) showed that 72±20% of cell stained for oligodendroglial marker (NG2 Chondroitin Sulfate Proteoglycan), 70±35% cell stained for neuronal marker (Neuronal class III beta tubulin) and 96±4% of cells stained for astroglial marker (Glial fibrillary Acidic Protein).

**Table 3:** NPC-derived tumor cell cultures (NPC-DTCC) characteristics.

	Pre Tx NPC	#1	#3	#3a	#8	#16	#1bis	#3bis
**Passage number**	≤p15	p8	p3	p4	p2	p4	p5	p7
**Cell cycle Analysis**								
- subG1	14	37	58	16	14	22	31	8
- G1	45	49	6	15	46	42	21	54
- S	7	1	-	2	5	4	3	3
- G2	32	11	11	27	13	30	27	20
- HyperG2	3	3	24	30	11	3	18	16
- G2/G1	0.71	0.22	1.7	1.9	0.3	0.7	1.3	0.4
**Karyotype**								
- chromosome§	40 (40/42)	40 (38/43)	39 (34/79)	66 (37/78)	43 (39/50)	42 (40/45)	59 (55/90)	53 (33/58)
- euploidia#	90% (18/20)	40% (8/20)	0% (0/9)	0% (0/19)	0% (0/12)	5% (1/20)	0% (0/15)	0% (0/15)
**Doubling time (h)**	72	38	48	45	94	85	51	48
**Clonogenicity** *	16.1	8.3	2.5	15.7	54.2	92	10.7	Nt
***In vivo*** ** tumorigenicity:**								
-Under skin (1×10^6^ cells)	0/8	3/3	3/3	3/3	3/3	3/3	3/3	3/3
-Kidney capsule (1×10^6^ cells)	0/8	3/3	3/3	3/3	3/3	3/3	3/3	3/3

§ data are expressed as number median (min/max).

# data are expressed as % of methaphases with 40 Chr and (n).

*expressed as number of cell for well to obtain clone development in 50% of wells (see Material and method); Nt =  not tested.

## Discussion

Athough pioneer *in vivo* studies with NPC focused mainly on their ability to promote structural and functional repair of damaged brain tissues owing to their ‘stem-cell’ properties, the data available on the immunomodulatory properties of NPC support their possible use as a modulator for immune-mediated process.

We tested the possibility to use NPC as cell therapy for tolerance induction in tissue transplantation outside the central nervous system. The immunomodulatory activity of NPCs has been previously described exclusively in experimental models of neurological disease (*i.e.* experimental autoimmune encephalomyelitis, intracerebral haemorrhage) [3]–[7], [9]–[11] and after systemic injection due to their interaction with immune cells in secondary lymphoid organs [8].

We demonstrate that a cell therapy approach that combines NPCs with islet transplant prevents acute islet allograft rejection, enriches CD4^+^CD25^+^FoxP3^+^ regulatory T cells in the spleen and induces active tolerance. On the other hand, this condition appears strictly associated with the development of NPC-derived cancers mainly sustained by insulin secretion.

These results introduce a new concept in the field of stem cell-based therapy and provide a new experimental model to study the stem-cell oncogenesis associated with immune escape. NPCs were previously described to display a strikingly stable profile with regard to self-renewal, expansion, differentiation, growth factor dependence, karyotype and molecular profiling. Foroni et al. reported that when forced to grow *in vitro* for over 100 passages, NPCs do not undergo transformation and never give rise to tumors *in vivo*, even when immortalized through delivery of *Myc* and *Ras* oncogenes [21]. A single previous report has proposed that the extensive culturing of NPCs [22]–[27] might select for aggressive cell clones but only after very long-term culture [27]. More generally, many studies strongly support the notion that spontaneous transformation of adult stem cells can take place exclusively after long-term culture *in vitro* [22]–[27]. Here, we demonstrate that adult multipotent neural stem/precursor cells can generate cancer *in vivo* even when used after short-term culture *in vitro*. In fact, we avoided using NPCs from neurospheres beyond 15 passages and NPCs appeared euploid with normal karyotype at time of injection. Despite this, 70% and 100% of mice transplanted with allogeneic or singeneic NPCs respectively unequivocally developed cancer. The malignant nature of these tumors was demonstrated by the histological morphology, by the presence of metastatic behaviour *in vivo* and by the expression of all the features of malignant cancer cells *in vitro* (i.e. capacity to extensive proliferation, unlimited self-renewal ability, loss of growth-factor dependence, clonogenicity, karyotype changes with chromosomal aberrations, aneuploidia, tumorigenic activity and multipotency).

The localization of NPCs with islets in the same microenvironment appears the “conditio sine qua non” for cancer development, since we did not observe tumors in NPCs transplanted alone both in allogeneic and syngeneic settings. This evidence introduces the concept that “safety” of stem cell therapy in terms of cancer generation is not only an intrinsic property of stem cells, but is also strictly dependent on cross talk with the microenvironment. In our model, insulin appears likely to be responsible for the malignant transformation of NPCs. Identification of the mechanisms by which insulin induces the NPC transformation was beyond the scope of this study and will be the matter of future work. However, we can speculate that insulin may modulate cancer development through both the insulin receptor and insulin like growth factor-1 receptor (IGF-1R). Insulin receptor activation by insulin triggers intracellular signalling cascades in both the extracellular-signal-regulated kinase (ERK) and phosphotidylinsositol-3 kinase (PI-3K) pathways, and thus, insulin signalling has the “machinery” to be mitogenic and anti-apoptotic [28]. Conventionally, however, it is thought that insulin is mitogenic only at supraphysiological levels and its main proliferative effects are probably mediated through the IGF-I-1R. Of note is the fact that both insulin receptor and IGF-1R were repeatedly shown to be associated with brain tumour development, proliferation and response to therapy in humans. The presence of an active IGF system has been established in glioblastoma [29], neuroblastomas [30], astrocytomas and meningiomas [31]. Similarly atypical teratoid/rhabdoid tumours of the CNS over-express the insulin receptor [32].

The localization of NPCs with islets in the same microenvironment appears the “conditio sine qua non” for preventing rejection, as demonstrated by the fact that mice transplanted with islets under the left kidney capsule and with NPCs under the right kidney capsule did not develop tolerance. Since graft survival was always associated with cancer development, the competence in inducing tolerance appears associated with oncogenic transformation rather than to an intrinsic property of NPCs. The mechanisms by which NPC oncogenic transformation protects islet from alloimmune attack is likely to be similar to those observed during drug-mediated tolerance induction. The histological appearance of the transplant (very low cellular infiltration and, when present, confined to the periphery of the islets) is consistent with that described previously during drug-mediated tolerance via expansion of regulatory T cells [20]. Over the last years a number of reports have described elevated numbers of in situ mechanisms able to block the immune reaction in tumor microenvironment, including the presence of regulatory T cells [33]–[35]. In agreement with this hypothesis, in our model, tolerance was associated with a significant reduction of total CD4^+^ T cells and with a concomitant expansion of CD4^+^CD25^+^FoxP3^+^ regulatory T cells in the spleen. Of note, patients with malignant tumors of central nervous system (i.e. gliomas) are characterized by dramatic reductions in CD4^+^ T cell number and a disproportionate presence of immunosuppressive regulatory T cells [36], exactly the same features of our mice bearing NPC-derived tumors. However, we cannot exclude that other mechanisms play a relevant role in preserving tumor embedded islets, including the release of local immunosuppressive factors, the inability of leukocytes to infiltrate neoplastic tissue and the presence of a generalized immune-compromised state due to the large tumor mass. At this time we do not know whether transplantation of tumor cells from any other cellular origin can produce results similar to those obtained with NPCs. It should also be considered that if there are identifiable specific immunomodulatory factors produced by the transformed NPCs that protects transplanted islets, this could have therapeutic implication, even if the NPCs cause tumors. Of note, the release of major developmental stem cell regulators, including the morphogens bone morphogenetic protein (BMP)-4 and sonic hedgehog (Shh), the extracellular matrix protein tenascin C, and the BMP antagonist Noggin have been reported to be responsible for immune regulatory activity of “normal” NPCs [8].

More generally, and beyond our original intention, transplantation of NPCs with islets offers an experimental model to study the oncogenesis associated with immune escape. In fact, NPC-derived cancer grows in fully mismatched allogenic recipients. Moreover cancer development is relatively fast, frequent and reproducible offering the possibility to study both the oncogenetic process in vivo at different stages (starting from non neoplastic NPC to malignant NPC-DTCC) and the associated mechanisms of immune escape.

In conclusion, our results demonstrated that in diabetic mice receiving allogeneic islet transplantation the concomitant administration of NPCs induce stable long-term islet function in the absence of immunosuppression and that this was not reversible by alloantigen re-challenge. This condition was associated with splenic expansion of CD4^+^/CD25^+^/FoxP3^+^ cell. Unfortunately, we also found constant and reproducible development of NPC derived cancer mainly sustained by the insulin secretion. Our result raises serious doubts about the safety of using adult stem cells outside the original microenvironment.

## Materials and Methods

### Ethics Statement

All animals were handled in strict accordance with good animal practice as defined by the relevant international (Directive 86/609/EEC and the recommendation 2007/526/EC from European community), national (Legislative Decree 116/92 and law n. 413/1993) and according to protocol approved by the Animal Care and Use Committee of the Fondazione San Raffaele del Monte Tabor (IACUC #280) and communicated to the Ministry of Health and local authorities according to the Italian law. As a general rule we applied all available methods to replace, reduce and refine animal use (Three Rs) in our experimental plan. All experiments were terminated 140 days after islet Tx or when significant sign of animal suffering became apparent. All interventions were performed under general anaesthesia if suffering had to be expected. All animal experiments were performed in our central animal facility by trained and experienced personnel. Accreditation from the local authorities has been obtained for all personnel involved in animal experiments.

### Islets isolation and culture

Pancreatic islets were isolated from C57BL/6 mice (nine weeks old, 20–22 g; Charles River, Calco, Italy) by a collagenase digestion method, as described [37]. Briefly, 2 ml of cold Hank's buffer/collagenase type V solution (1 mg/ml; Sigma, St Louis, MS, USA) was infused into the pancreatic duct *in situ*, and the removed pancreas was digested at 37°C for 15 min. Islets were purified on a discontinuous Ficoll gradient (Sigma). The islets (250 islet/ml) were cultured free-floating (37°C, 5% CO_2_) in medium RPMI 1640 (Bio-Whittaker, Walkersville, MD, USA) supplemented with L-glutamine (Sigma), penicillin-streptomycin (1000 U/ml-10 mg/ml; Sigma) and 10% (vol/vol) fetal calf serum (HyClone, Celbio, Logan, UT, USA) for 20–24 h before the transplant. Islet purity was >90%.

### NPC derivation and cultures

Adult neurospheres were generated from the SVZ of four-to-eight week-old C57Bl/6, as described [8]. Mice were anesthetized by intraperitoneal injection of pentobarbital (120 mg/kg) and killed by cervical dislocation. The brains were removed and placed in artificial cerebrospinal fluid (aCSF) (124 mM NaCl, 5 mM KCl, 1.3 mM MgCl2, 0.1 mM CaCl2, 26 mM NaHCO3, and 10 mM D-glucose, pH 7.3) aerated with 95% O2/5% CO2 at room temperature. The SVZ neural tissue – excluding the subependyma – was isolated after coronal sectioning and cut into 1 mm^3^ pieces. Pieces were transferred into 30 ml of aCSF containing 1.3 mg/ml trypsin, 0.67 mg/ml hyaluronidase, and 0.2 mg/ml kynurenic acid (all from Sigma) and incubated, under continuous oxygenation and stirring, for 90 min at 32–34°C. Tissue sections were then rinsed in aCSF for 10 min, transferred to DMEM/F12 (Life Technologies, Rockville, MD) medium containing 0.7 mg/ml ovomucoid (Sigma), and carefully triturated with a fire-polished Pasteur pipette. Cells were collected by centrifugation and re-suspended in GF-free, chemically defined DMEM/F12 medium containing 2 mM L-glutamine, 0.6% glucose, 9.6 mg/ml putrescine, 6.3 ng/ml progesterone, 5.2 ng/ml sodium selenite, 0.025 mg/ml insulin, 0.1 mg/ml transferrin, and 2 µg/ml heparin (Sigma). Cells were then cultured in NeuroCult® Proliferation Kit (Stem Cell Technologies, Vancouver BC, Canada). The number of primary spheres was counted after 7–12 days in vitro (DIV). For cell amplification, 8000 cells/cm^2^ were plated at each sub-culturing passage in untreated tissue culture flasks. After 3–4 days (time estimated to obtain the doubling of cell number), neurospheres were harvested, mechanically dissociated, counted and re-plated under the same culture conditions. NPCs at passage number ≤15 were used in all experiments. Cells were labelled *in vitro* using a lentiviral vector containing the enhanced green fluorescent protein (GFP).

### Islets and NPC transplantation

Male Balb/c mice (nine week old, 20–22 g; from Charles River) were used as recipients. Mice were made diabetic (non-fasting blood glucose levels between 400 and 600 mg/dl) with intravenous STZ injection (175 to 200 mg/kg; Sigma) one week before transplantation. The recipient mice were anesthetized with isoflurane (Forane 1–2%; Abbott Laboratories, Toronto, Canada). Islets were transplanted as previously described [37]. Briefly, after exposure of the left kidney, P-50 tubing containing the islets was inserted through a small incision into the lower pole of the kidney and pushed to the upper pole, whereupon the islets were gently pushed beneath the kidney capsule, the cannula was removed, and the incision was cauterized. The following four groups of recipients were used: group 1 received a pure islet graft (350 equivalent islets [EI]; n = 12); group 2 received a mixed islet/NPC graft (1×10^3^ NPCs as neurospheres plus 350 equivalent islets under the capsule of the left kidney; n = 10); group 3 received islets and NPC graft (1×10^3^ NPCs as neurospheres under the capsule of right kidney, 350 equivalent islets under the capsule of the left kidney; n = 7); group 4 received a pure islet graft in the presence of immunosuppression (350 equivalent islets; n = 26). As immunosuppression we used the Edmonton protocol consisting of rapamycin plus FK506 plus anti–IL-2Rα chain mAbs [38]. The treatment of the transplanted Balb/c mice began the day after transplant. Rapamycin (Rapamune; Wyeth-Ayerst, Pearl River, NY) was diluted in peanut oil (Sigma) and administered once daily for 30 consecutive days at a dose of 1 mg/kg by gavage. FK506 (Prograf; Fujisawa) was diluted in saline solution and administered once daily for 30 consecutive days at 0.3 mg/kg, i.p. Anti–mouse IL-2Rα chain mAb (clone 7D4; BD Biosciences, Mountain View, CA) was diluted in saline solution and administered intraperitoneally at time 0 and 4 days after islet transplantation to reach a dose of 1 mg/mouse. Blood glucose levels were measured using a Glucometer Elite (Bayer Canada, Toronto, Ontario, Canada) at 15, 30 and 60 minutes after the end of surgical procedure, daily for the first week and then every second day post-transplantation. The animals had free access to tap water and pelleted food throughout the course of the study. The local animal ethics committee approved all experiments. Surgical death was defined as death within the first 7 days after transplantation. Primary graft non function was defined as the inability to reach non-fasting blood glucose levels under 250 mg/dl for two consecutive measurement after islet transplantation, and rejection was defined as two successive measurements >300 mg/dl in mice where primary function was achieved. The Balb/c mice that did not reject the islet allograft 100 days after transplantation were boosted *in vivo* with allogeneic splenocytes [20]. A total of 30×10^6^ splenocytes isolated from C57BL/6 mice were injected intraperitoneally. Blood glucose levels were monitored daily thereafter for 40 days, then mice were killed, transplant and spleen were collected.

### Sustained Release Insulin Implants and NPC transplantation

To specifically test the role of insulin on NPC, in some experiments we substituted islets with an insulin releasing pellet. After exposure of the left kidney, P-50 tubing containing NPC was inserted through a small incision into the lower pole of the kidney and pushed to the upper pole, whereupon the cells were gently pushed beneath the kidney capsule, the cannula was removed. Then we pushed a 2×3 mm insulin release pellet (LinBit for mice, Linshin Canada. Inc. Ontario, Canada) through the capsule orifice just created from the cannula and the incision was cauterized. Insulin release rate from the pellet is ∼0.1 U/24 hr/implant for >30 days. The implant dose has enough insulin to last for >40 days at the specified release rate The remnants of the insulin-depleted implants are totally absorbed in the next weeks, and removal is not necessary.

### Preparation and analysis of spleen mononuclear cells

Cell culture medium was prepared with RPMI 1640 (Bio-Whittaker, Walkersville, MD, USA) supplemented with 1000 U/ml penicillin-streptomycin (Sigma) and 10% (vol/vol) FCS (fetal calf serum, HyClone, Celbio, Logan, UT, USA). Spleen from transplanted and non-transplanted control mice were harvested and processed within 2 h mouse sacrifice. Tissues were first homogenized in sterile 0,4 µm cell strainers to remove large debris. To lyses erythrocytes, the cell suspension was resuspended in 1 ml of sterile water and immediately washed in cell culture medium. Cell staining and flow cytometry analysis staining buffer was prepared with PBS (Phosphate buffered saline, without Ca2+ and Mg2+, EuroClone Ltd., Torquay, UK) supplemented with 2,5% (vol/vol) FCS and 0,01% (vol/vol) sodium azide. All monoclonal antibodies specific for surface antigen were directly conjugated and purchased from BD pharmingen (San Jose, California). No tandem-dye-conjugated monoclonal antibodies were used. Intracellular staining for FoxP3 was performed with FITC-conjugated anti-mouse FoxP3 (clone FJK-16s) and FoxP3-staining kit (both from eBioscience San Diego, California). For each staining a total of 3×10^5^ splenocytes were incubated 30 min at 4°C with the respective monoclonal antibodies properly diluted in staining buffer. Cells were than washed and resuspended in 200 µl of staining buffer and acquired within 24 hours. Intracellular staining for FoxP3 was performed with the kit-provided buffers and following the suggested protocol. All samples were acquired on a CyAn ADP (Beckman Coulter, Brea, California, USA) or on a FacsCanto II (BD Biosciences) instrument. Flow cytometry data analysis was performed with Summit ver 4.3 (Beckman Coulter, Brea, California, USA) or Fcsexpress ver 3.0 (De Novo Software).

### Characterization of harvested grafts

For morphological investigation the recipients were sacrificed 140 days after transplantation: the kidney was removed and fixed in 10% buffered formalin and processed for histology. Histologic sections were stained with hematoxylin and eosin (H&E) or for insulin with the use of an anti-insulin guinea pig primary polyclonal antibody (Dako, Carpinteria, CA, USA). The streptavidin-biotin method (LSAB-2 Kit; Dako) or AffiniPure anti–guinea pig tetramethylrhodamine-5 (and 6)-isothiocyanate (TRITC) secondary antibodies (Jackson ImmunoResearch Laboratories) were used for detection.

### NPC-derived tumor cell cultures (NPC-DTCC), culture propagation, population analysis, and cloning

Seven primary cell cultures were established from NPC-derived tumor. Cells were plated at a density of 8,000/cm^2^ in RPMI 1640 (Bio-Whittaker) supplemented with L-glutamine (Sigma), penicillin-streptomycin (1000 U/ml-10 mg/ml; Sigma) and 10% (vol/vol) fetal calf serum (HyClone). For growth curves, 200,000 cells were replated in a 25-cm^2^ flask after each subculturing passage. Population and serial clonal analyses were done as described previously [21]. Cell cycle analysis was performed by propidium iodide (Sigma) staining of permeabilized cells. Clonal efficiency was assessed using a limiting dilution assay. Briefly, 0.1 to 50 cells per round- bottomed well (96-well plate) were incubated in 100 µl RPMI 1640 (Bio-Whittaker) containing 10% FCS. Twelve wells were seeded per cell concentration. After 14 days of culture, positive wells were scored using an inverted microscope. Clonal efficiency was quantified as the cell concentration able to obtain clones development in 50% of wells. Tumorigenicity of NPC-TDCC was determined by injecting 1×10^6^ cells at passages 5 either s.c. or under the kidney capsule of 12-week-old syngeneic C57BL/6 mice. Chromosome analysis was done as previously described [21]. Briefly, cells were treated with medium containing 10 µg/mL Colcemid (Irvine Scientific, Santa Ana, CA) for 1 to 2 hours and resuspended in hypotonic 1% sodium citrate at room temperature for 30 minutes. The cells were then washed in methanol-acetic acid (3∶1, v/v) fixative solution for 30 minutes and spread onto clean dry slides. Q-banding staining was then performed, and 9 to 20 metaphases were analyzed for each sample. Immunofluorescence for neural antigens was done as previously described [39]. Mouse monoclonal antibody against Neuronal Class III β-Tubulin (Covance, California), polyclonal rabbit anti-Glial Fibrillary Acid Protein (Dako, Carpinteria, CA, USA) and polyclonal rabbit anti-NG2 Chondroitin Sulfate Proteoglycan (Chemicon, USA) were used for the immunostaining. AffiniPure anti–mouse or anti-rabbit tetramethylrhodamine-5 (and 6)-isothiocyanate (TRITC) secondary antibodies (Jackson ImmunoResearch Laboratories) were used for detection. Sections were mounted in VECTASHIELD® Mounting Medium with DAPI (Vector Lab, CA). Quantification of positive cells was performed with IN Cell Workstation 3.5 software.

### Statistical analysis

Significance of difference between groups was tested using the Mann-Whitney U test, Kruskal Wallis test, Pearson chi-square tests and One Way ANOVA test. Survival differences were tested using the log rank statistic. For all analyses, a two-tailed P value of 0.05 was considered significant. Statistical analyses were performed using the Statistical Package for Social Science (SPSS 13.0; SPSS, Chicago, IL).

## Supporting Information

Figure S1 NPC/islet co-transplantation and co-localization induces tumor formation. Diabetic Balb/c mice were transplanted under the kidney capsule with 350 equivalent islets purified from C57BL/6 mice. Islets were transplanted alone under the left kidney capsule (Islet-Tx) or co-transplanted with GFP+NPC (1,000 neurospheres) alternatively in controlateral right kidney (NPC-Tx) or co-localized in the left kidney (Co-NPC-Tx). The mice surviving until day 140 were sacrificed. A laparotomy to evaluate the graft-bearing kidney was performed. No residual macroscopic or microscopic grafted tissue (islet or GFP+NPC) were recognized at the level of the kidney capsules in Islet-Tx and NPC-Tx (respectively Panel A and B). On the other hand, in 7/10 of the Co-NPC-Tx tumor mass substituted the kidney parenchyma (Panel C. Upper: case #1; lower: case #3; see table 2). After tissue perfusion and washing tumor were analyzed for GFP expression using inverted fluorescent microscopy. The tumoral tissue showed a strong positivity for GFP. Black arrow: left kidney.
(0.31 MB PDF)

Figure S2 NPC/insulin releasing pellet co-transplantation and co-localization induces tumor formation. Three diabetic C57BL/6 mice were transplanted with syngeneic NPC (1,000 neurospheres) and sustained release insulin implants (release rate: ∼0.1 U/24 hr/implant for >30 days) under the left kidney capsule and with syngeneic NPC alone (1,000 neurospheres) under the right kidney capsule. Panel A: not fasting blood glucose profile after receiving NPC/insulin releasing pellet co-transplantation. Data are expressed as mean (line) and ±1 standard deviation (area). Panel B: morphological appearances of the haematoxylin and eosin stained kidneys. Histological appearance (1x, insert 20x) of NPC/insulin releasing pellet (upper) and NPC alone (lower) in a representative C57BL/6 mouse 140 days after transplantation.
(0.82 MB PDF)

Figure S3 Malignant features of NPC-derived tumor. (A) NPC-derived tumor infiltrating adjacent kidney (H&E; 10x); (B) Ki-67 staining on NPC- derived tumors (4x).
(0.81 MB PDF)

Figure S4 NPC-derived tumor cell cultures (NPC-DTCC). NPC-DTCC derived from tumors of mice co-transplanted with islet and GFP+ NPC. All the cell in culture were and maintained the positivity for GFP, demonstrating their original derivation from NPC. Left: phase contrast image at passage 16; Right: fluorescent image of the same field showing GFP+. Scale bar: 100 µm.
(1.31 MB PDF)

Figure S5 Proliferation analysis of NPC-derived tumor cell cultures (NPC-DTCC). Panel A: long-term proliferation curves for the 7 established primary cell cultures. The total number of cells cultured for >200 days *in vitro* was calculated at each subculturing passage. Exponential expansion rate was maintained over time and the total amount of cells, yielded from a starting number of 40×103, was 1050 cells for each cell line. Panel B: Relative increase of NPC-DTCC growth rate was assessed by plotting the number of cells yielded at each single subculturing passage (from 16 to 32 passages). Curves reveal a modest but constant upward trend of proliferation rate throughout long-term culturing.
(0.01 MB PDF)

Table S1 Tumor formation after islet/NPC co-transplantation in allogenic and singeneic models.
(0.05 MB DOC)

## References

[1] Martino G, Pluchino S (2006). The therapeutic potential of neural stem cells.. Nat Rev Neurosci.

[2] Miller RH (2006). The promise of stem cells for neural repair.. Brain Res.

[3] Pluchino S, Zanotti L, Rossi B, Brambilla E, Ottoboni L (2005). Neurosphere-derived multipotent precursors promote neuroprotection by an immunomodulatory mechanism.. Nature.

[4] Pluchino S, Quattrini A, Brambilla E, Gritti A, Salani G (2003). Injection of adult neurospheres induces recovery in a chronic model of multiple sclerosis.. Nature.

[5] Ben-Hur T, Einstein O, Mizrachi-Kol R, Ben-Menachem O, Reinhartz E (2003). Transplanted multipotential neural precursor cells migrate into the inflamed white matter in response to experimental autoimmune encephalomyelitis.. Glia.

[6] Einstein O, Karussis D, Grigoriadis N, Mizrachi-Kol R, Reinhartz E (2003). Intraventricular transplantation of neural precursor cell spheres attenuates acute experimental allergic encephalomyelitis.. Mol Cell Neurosci.

[7] Einstein O, Fainstein N, Vaknin I, Mizrachi-Kol R, Reihartz E (2007). Neural precursors attenuate autoimmune encephalomyelitis by peripheral immunosuppression.. Ann Neurol.

[8] Pluchino S, Zanotti L, Brambilla E, Rovere-Querini P, Capobianco A (2009). Immune regulatory neural stem/precursor cells protect from central nervous system autoimmunity by restraining dendritic cell function.. PLoS One.

[9] Lee ST, Chu K, Jung KH, Kim SJ, Kim DH (2008). Anti-inflammatory mechanism of intravascular neural stem cell transplantation in haemorrhagic stroke.. Brain.

[10] Wang L, Shi J, van Ginkel FW, Lan L, Niemeyer G (2009). Neural stem/progenitor cells modulate immune responses by suppressing T lymphocytes with nitric oxide and prostaglandin E2.. Exp Neurol.

[11] Fainstein N, Vaknin I, Einstein O, Zisman P, Ben Sasson SZ (2008). Neural precursor cells inhibit multiple inflammatory signals.. Mol Cell Neurosci.

[12] Lu Z, Hu X, Zhu C, Wang D, Zheng X (2009). Overexpression of CNTF in Mesenchymal Stem Cells reduces demyelination and induces clinical recovery in experimental autoimmune encephalomyelitis mice.. J Neuroimmunol.

[13] Zappia E, Casazza S, Pedemonte E, Benvenuto F, Bonanni I (2005). Mesenchymal stem cells ameliorate experimental autoimmune encephalomyelitis inducing T-cell anergy.. Blood.

[14] Escolar ML, Poe MD, Provenzale JM, Richards KC, Allison J (2005). Transplantation of umbilical-cord blood in babies with infantile Krabbe's disease.. N Engl J Med.

[15] Robertson RP (2004). Islet transplantation as a treatment for diabetes - a work in progress.. N Engl J Med.

[16] Marzorati S, Pileggi A, Ricordi C (2007). Allogeneic islet transplantation.. Expert Opin Biol Ther.

[17] Solari MG, Srinivasan S, Boumaza I, Unadkat J, Harb G (2009). Marginal mass islet transplantation with autologous mesenchymal stem cells promotes long-term islet allograft survival and sustained normoglycemia.. J Autoimmun.

[18] Sordi V, Melzi R, Mercalli A, Formicola R, Doglioni C (2010). Mesenchymal cells appearing in pancreatic tissue culture are bone marrow-derived stem cells with the capacity to improve transplanted islet function.. Stem Cells.

[19] Mineo D, Ricordi C, Xu X, Pileggi A, Garcia-Morales R (2008). Combined islet and hematopoietic stem cell allotransplantation: a clinical pilot trial to induce chimerism and graft tolerance.. Am J Transplant.

[20] Battaglia M, Stabilini A, Draghici E, Gregori S, Mocchetti C (2006). Rapamycin and interleukin-10 treatment induces T regulatory type 1 cells that mediate antigen-specific transplantation tolerance.. Diabetes.

[21] Foroni C, Galli R, Cipelletti B, Caumo A, Alberti S (2007). Resilience to transformation and inherent genetic and functional stability of adult neural stem cells ex vivo.. Cancer Res.

[22] Miura M, Miura Y, Padilla-Nash HM, Molinolo AA, Fu B (2006). Accumulated chromosomal instability in murine bone marrow mesenchymal stem cells leads to malignant transformation.. Stem Cells.

[23] Burns JS, Abdallah BM, Guldberg P, Rygaard J, Schroder HD (2005). Tumorigenic heterogeneity in cancer stem cells evolved from long-term cultures of telomerase-immortalized human mesenchymal stem cells.. Cancer Res.

[24] Rubio D, Garcia-Castro J, Martin MC, de la Fuente R, Cigudosa JC (2005). Spontaneous human adult stem cell transformation.. Cancer Res.

[25] Shiras A, Chettiar ST, Shepal V, Rajendran G, Prasad GR (2007). Spontaneous transformation of human adult nontumorigenic stem cells to cancer stem cells is driven by genomic instability in a human model of glioblastoma.. Stem Cells.

[26] Reynolds BA, Rietze RL (2005). Neural stem cells and neurospheres–re-evaluating the relationship.. Nat Methods.

[27] Morshead CM, Benveniste P, Iscove NN, van der Kooy D (2002). Hematopoietic competence is a rare property of neural stem cells that may depend on genetic and epigenetic alterations.. Nat Med.

[28] van der Heide LP, Ramakers GM, Smidt MP (2006). Insulin signaling in the central nervous system: learning to survive.. Prog Neurobiol.

[29] Trojan J, Johnson TR, Rudin SD, Ilan J, Tykocinski ML (1993). Treatment and prevention of rat glioblastoma by immunogenic C6 cells expressing antisense insulin-like growth factor I RNA.. Science.

[30] Martin DM, Yee D, Carlson RO, Feldman EL (1992). Gene expression of the insulin-like growth factors and their receptors in human neuroblastoma cell lines.. Brain Res Mol Brain Res.

[31] Antoniades HN, Galanopoulos T, Neville-Golden J, Maxwell M (1992). Expression of insulin-like growth factors I and II and their receptor mRNAs in primary human astrocytomas and meningiomas; in vivo studies using in situ hybridization and immunocytochemistry.. Int J Cancer.

[32] Arcaro A, Doepfner KT, Boller D, Guerreiro AS, Shalaby T (2007). Novel role for insulin as an autocrine growth factor for malignant brain tumour cells.. Biochem J.

[33] Beyer M, Schultze JL (2009). Regulatory T cells: major players in the tumor microenvironment.. Curr Pharm Des.

[34] Curiel TJ, Coukos G, Zou L, Alvarez X, Cheng P (2004). Specific recruitment of regulatory T cells in ovarian carcinoma fosters immune privilege and predicts reduced survival.. Nat Med.

[35] Liyanage UK, Moore TT, Joo HG, Tanaka Y, Herrmann V (2002). Prevalence of regulatory T cells is increased in peripheral blood and tumor microenvironment of patients with pancreas or breast adenocarcinoma.. J Immunol.

[36] Fecci PE, Mitchell DA, Whitesides JF, Xie W, Friedman AH (2006). Increased regulatory T-cell fraction amidst a diminished CD4 compartment explains cellular immune defects in patients with malignant glioma.. Cancer Res.

[37] Melzi R, Battaglia M, Draghici E, Bonifacio E, Piemonti L (2007). Relevance of hyperglycemia on the timing of functional loss of allogeneic islet transplants: implication for mouse model.. Transplantation.

[38] Shapiro AM, Ricordi C, Hering BJ, Auchincloss H, Lindblad R (2006). International trial of the Edmonton protocol for islet transplantation.. N Engl J Med.

[39] Gritti A, Parati EA, Cova L, Frolichsthal P, Galli R (1996). Multipotential stem cells from the adult mouse brain proliferate and self-renew in response to basic fibroblast growth factor.. J Neurosci.

