# Supplementary figures and images for: Co-Graft of Allogeneic Immune Regulatory Neural Stem Cells (NPC) and Pancreatic Islets Mediates Tolerance, while Inducing NPC-Derived Tumors in Mice
Source: PLoS One. 2010 Apr 27;5(4):e10357. doi: 10.1371/journal.pone.0010357 (PMC2860511; doi:10.1371/journal.pone.0010357)

**A**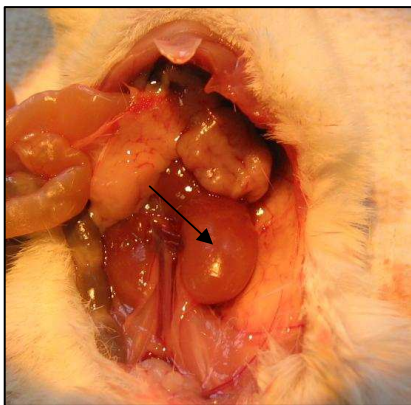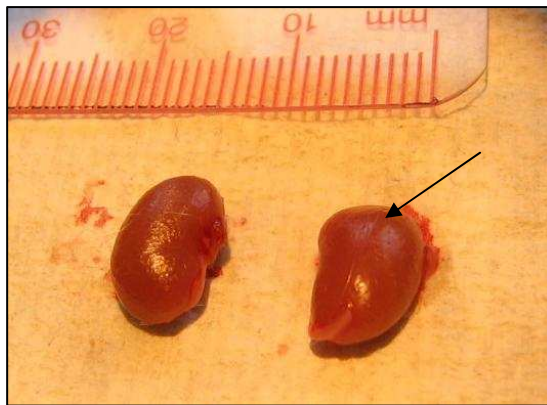**B**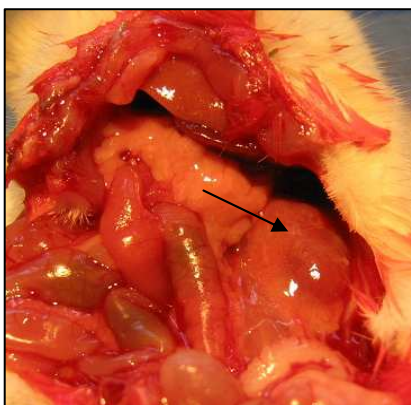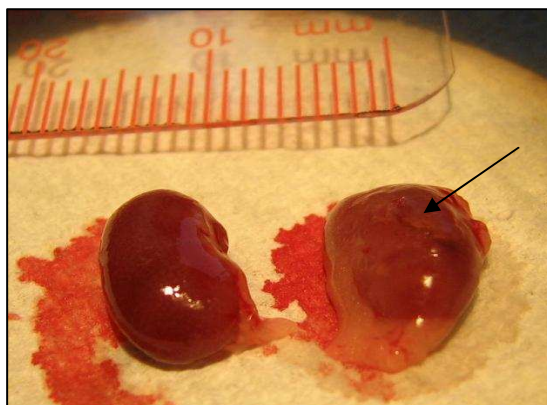**C**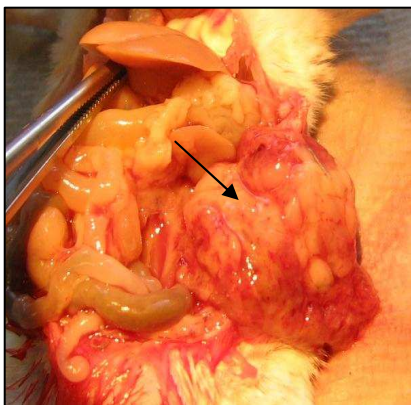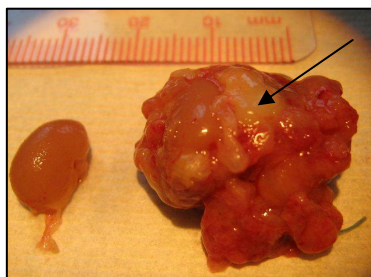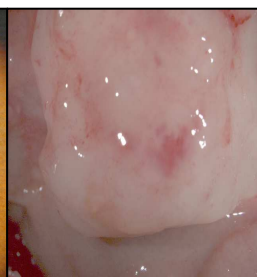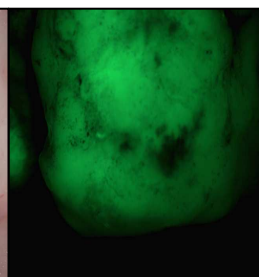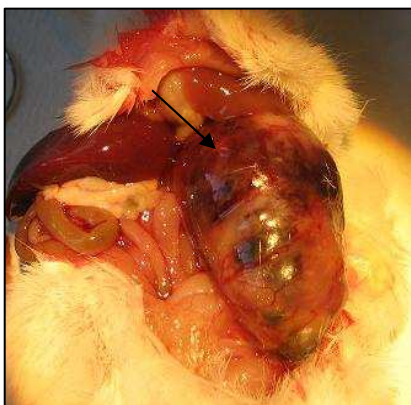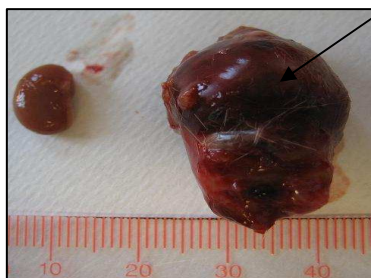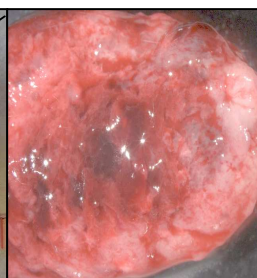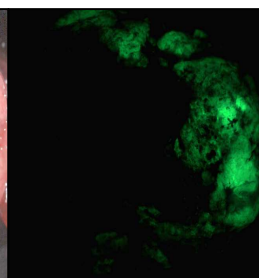

Fig S1

Supplement: Figure S1 — NPC/islet co-transplantation and co-localization induces tumor formation. Diabetic Balb/c mice were transplanted under the kidney capsule with 350 equivalent islets purified from C57BL/6 mice. Islets were transplanted alone under the left kidney capsule (Islet-Tx) or co-transplanted with GFP+NPC (1,000 neurospheres) alternatively in controlateral right kidney (NPC-Tx) or co-localized in the left kidney (Co-NPC-Tx). The mice surviving until day 140 were sacrificed. A laparotomy to evaluate the graft-bearing kidney was performed. No residual macroscopic or microscopic grafted tissue (islet or GFP+NPC) were recognized at the level of the kidney capsules in Islet-Tx and NPC-Tx (respectively Panel A and B). On the other hand, in 7/10 of the Co-NPC-Tx tumor mass substituted the kidney parenchyma (Panel C. Upper: case #1; lower: case #3; see table 2). After tissue perfusion and washing tumor were analyzed for GFP expression using inverted fluorescent microscopy. The tumoral tissue showed a strong positivity for GFP. Black arrow: left kidney. (0.31 MB PDF) [file pone.0010357.s001.pdf]

**A**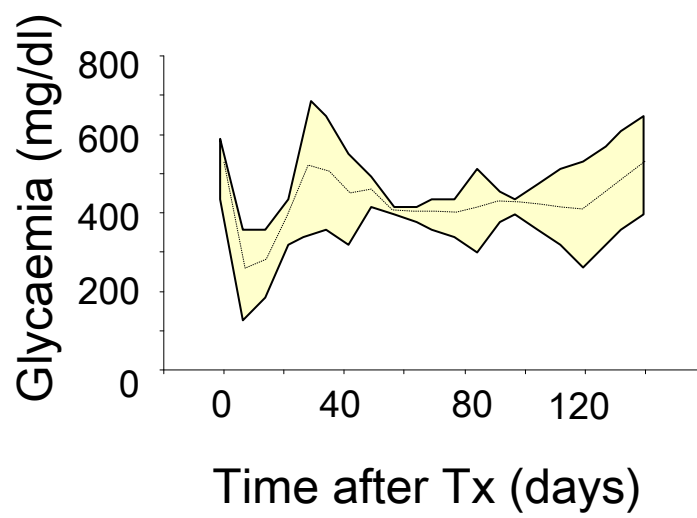**B**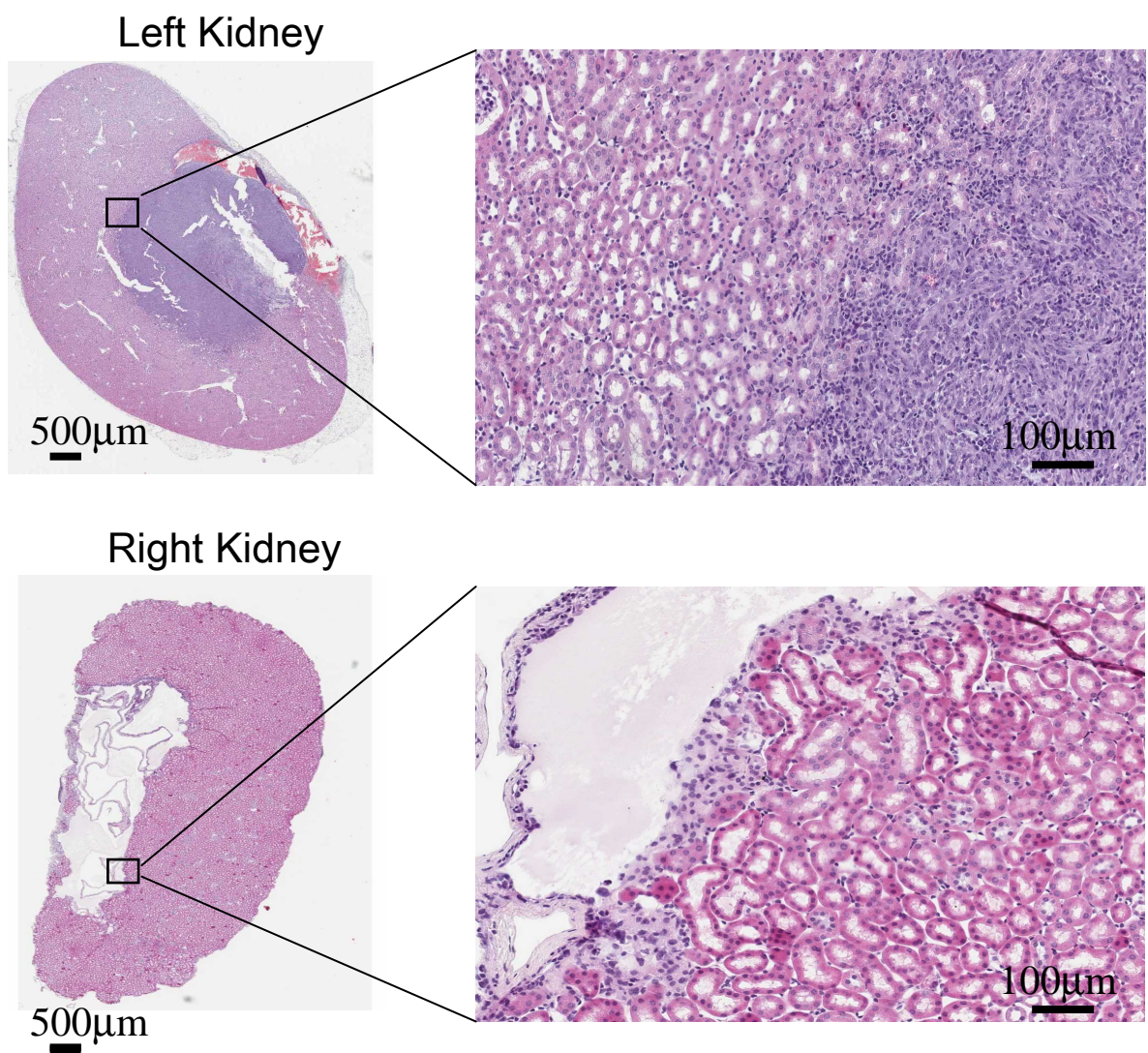**Fig S2**

Supplement: Figure S2 — NPC/insulin releasing pellet co-transplantation and co-localization induces tumor formation. Three diabetic C57BL/6 mice were transplanted with syngeneic NPC (1,000 neurospheres) and sustained release insulin implants (release rate: ∼0.1 U/24 hr/implant for >30 days) under the left kidney capsule and with syngeneic NPC alone (1,000 neurospheres) under the right kidney capsule. Panel A: not fasting blood glucose profile after receiving NPC/insulin releasing pellet co-transplantation. Data are expressed as mean (line) and ±1 standard deviation (area). Panel B: morphological appearances of the haematoxylin and eosin stained kidneys. Histological appearance (1x, insert 20x) of NPC/insulin releasing pellet (upper) and NPC alone (lower) in a representative C57BL/6 mouse 140 days after transplantation. (0.82 MB PDF) [file pone.0010357.s002.pdf]

**A**

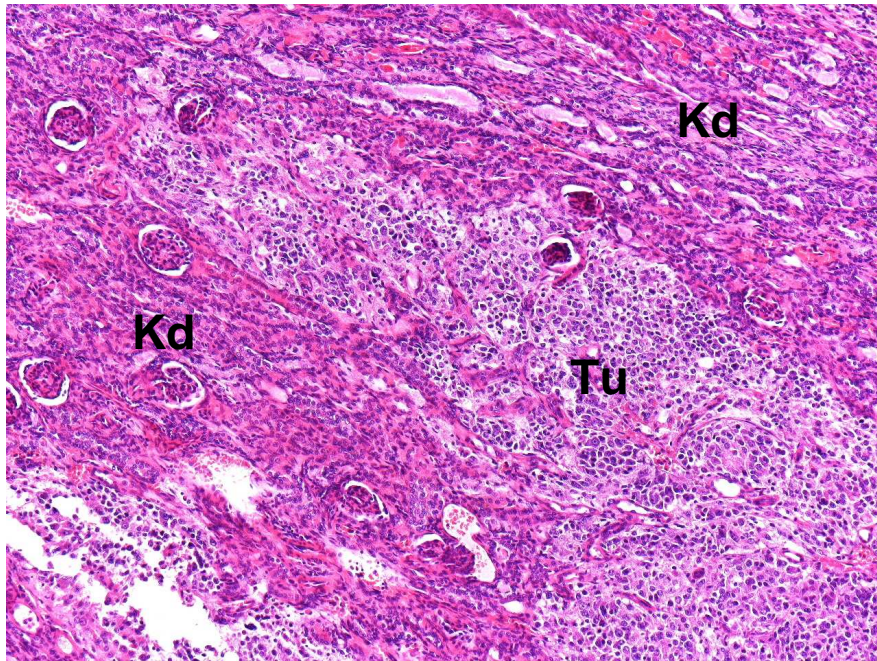

**B**

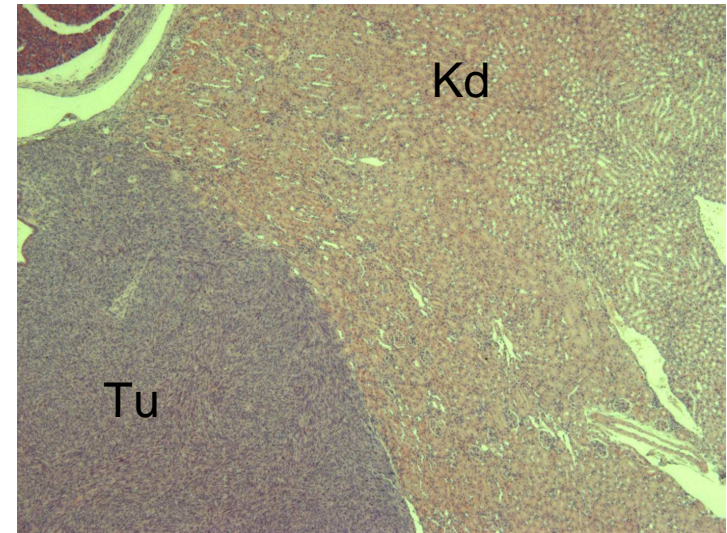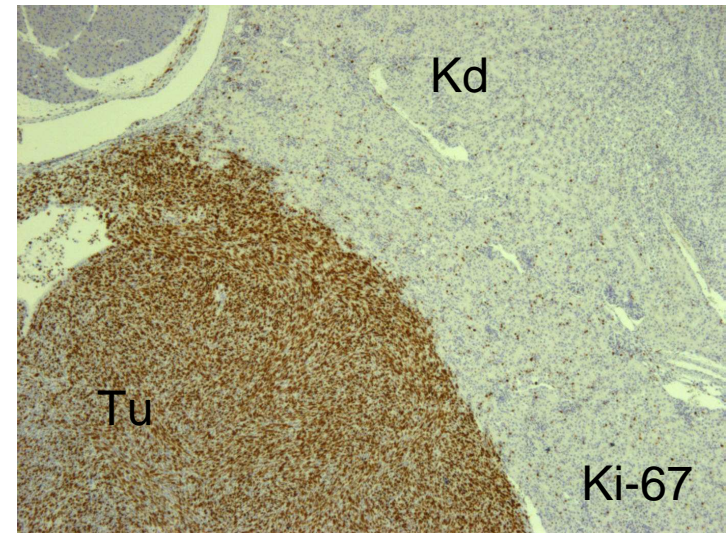

Fig S3

Supplement: Figure S3 — Malignant features of NPC-derived tumor. (A) NPC-derived tumor infiltrating adjacent kidney (H&E; 10x); (B) Ki-67 staining on NPC- derived tumors (4x). (0.81 MB PDF) [file pone.0010357.s003.pdf]

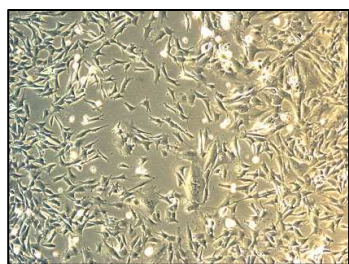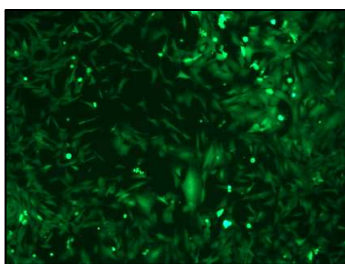

#1

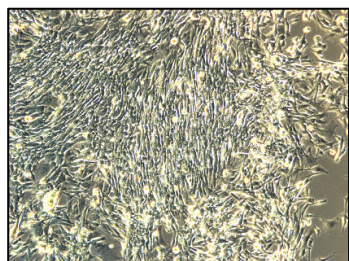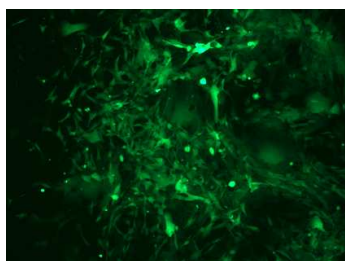

#3

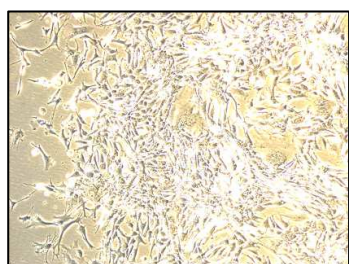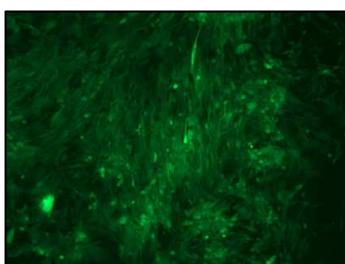

#3a

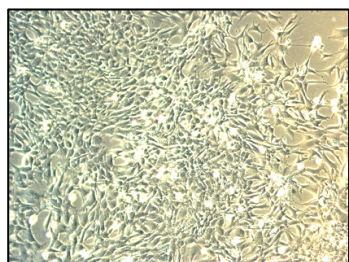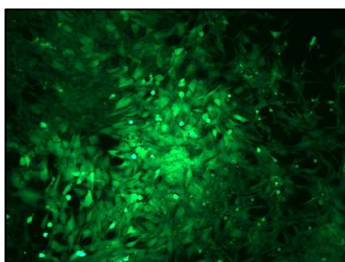

#8

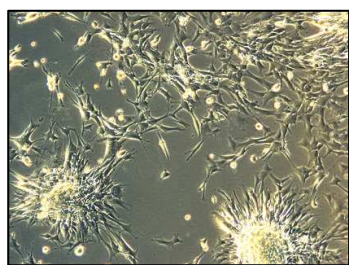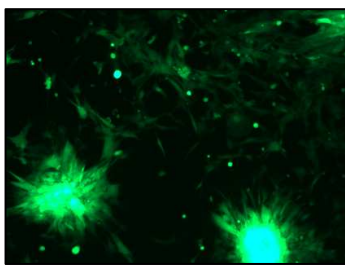

#16

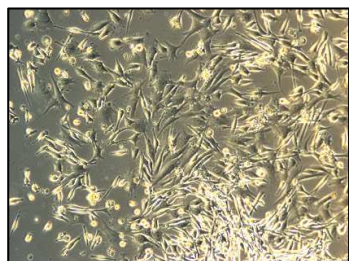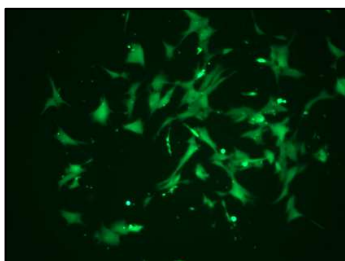

#1bis

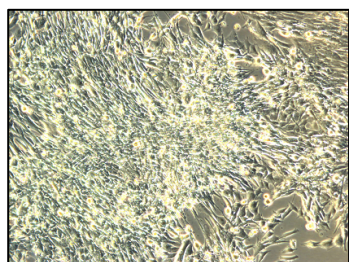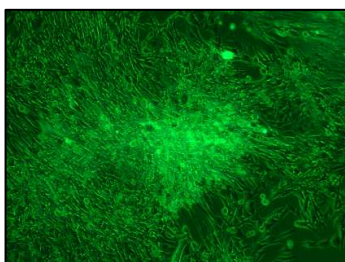

#3bis

Fig S4

Supplement: Figure S4 — NPC-derived tumor cell cultures (NPC-DTCC). NPC-DTCC derived from tumors of mice co-transplanted with islet and GFP+ NPC. All the cell in culture were and maintained the positivity for GFP, demonstrating their original derivation from NPC. Left: phase contrast image at passage 16; Right: fluorescent image of the same field showing GFP+. Scale bar: 100 µm. (1.31 MB PDF) [file pone.0010357.s004.pdf]

**A**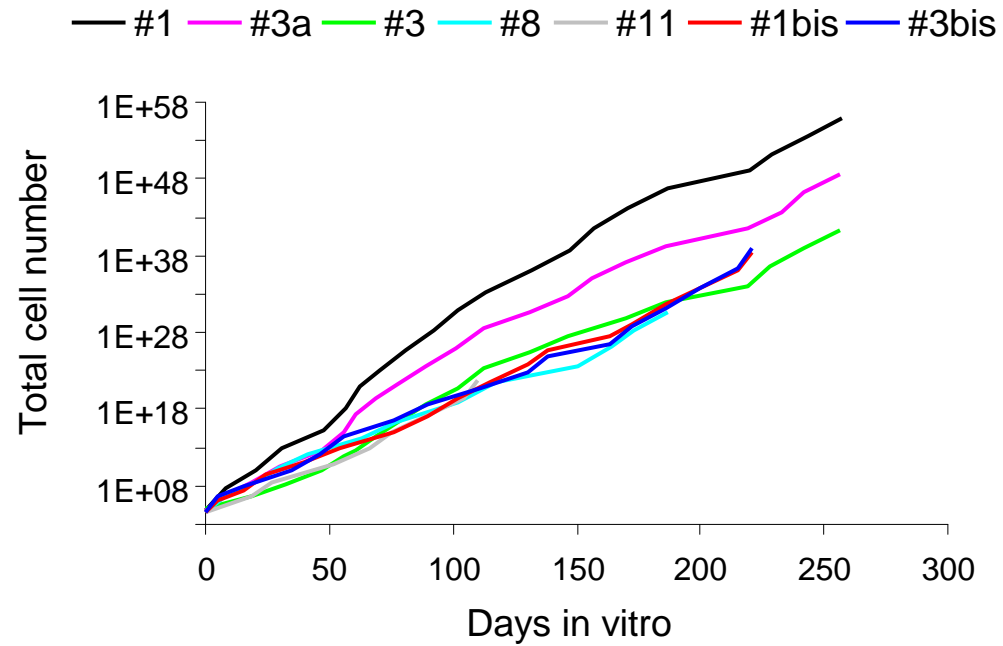**B**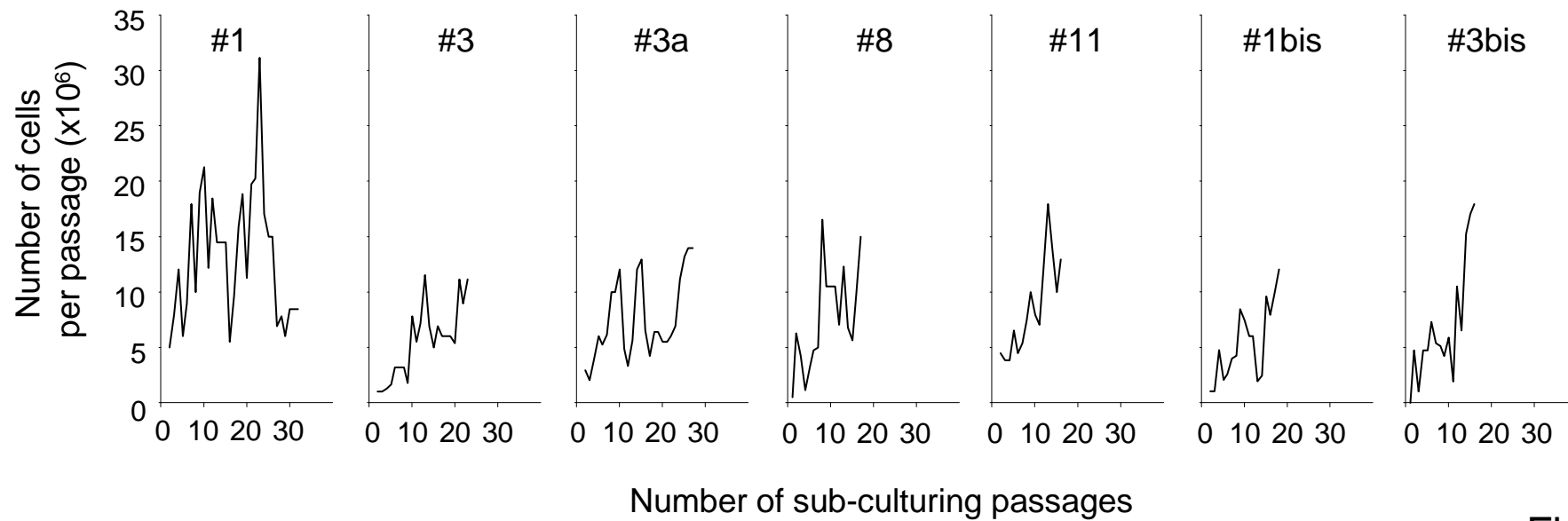**Fig S5**

Supplement: Figure S5 — Proliferation analysis of NPC-derived tumor cell cultures (NPC-DTCC). Panel A: long-term proliferation curves for the 7 established primary cell cultures. The total number of cells cultured for >200 days in vitro was calculated at each subculturing passage. Exponential expansion rate was maintained over time and the total amount of cells, yielded from a starting number of 40×103, was 1050 cells for each cell line. Panel B: Relative increase of NPC-DTCC growth rate was assessed by plotting the number of cells yielded at each single subculturing passage (from 16 to 32 passages). Curves reveal a modest but constant upward trend of proliferation rate throughout long-term culturing. (0.01 MB PDF) [file pone.0010357.s005.pdf]
