# Supplementary material for: Co-Graft of Allogeneic Immune Regulatory Neural Stem Cells (NPC) and Pancreatic Islets Mediates Tolerance, while Inducing NPC-Derived Tumors in Mice
Source: PLoS One. 2010 Apr 27;5(4):e10357. doi: 10.1371/journal.pone.0010357 (PMC2860511; doi:10.1371/journal.pone.0010357)
Supplement: Table S1 — Tumor formation after islet/NPC co-transplantation in allogenic and singeneic models. (0.05 MB DOC) [file pone.0010357.s006.doc]

**TABLE S1. Tumor formation after islet/NPC co-transplantation in allogenic and singeneic models.**

| Mouse ID | Islet function * | | Tumor# | | Metastasis | Primary culture |
| --- | --- | --- | --- | --- | --- | --- |
|  | After 100 d | After rechallenge | Left Kidney (islet/NPC) | Right Kidney (NPC) |  |  |
| Allogeneic model |  |  |  |  |  |  |
| #1 | yes | yes | yes (9.19) | - | Peritoneum, ascites | yes |
| #2 | yes | yes | yes (<0.1) | - | no | no |
| #3 | yes | yes | yes (14.4) | - | Peritoneum, liver, ascites | yes |
| #4 | yes | yes | yes (5.9) | - | no | no |
| #5 | no (88 ) | - | no | - | - | - |
| #6 | yes | no (9 ) | yes (0.4) | - | no | no |
| #7 | yes | no (6 ) | yes (<0.1) | - | no | no |
| #8 | yes | yes | yes (1.84) | - | no | yes |
| #9 | no (13 ) | - | no | - | - | - |
| #10 | no (22 ) | - | no | - | - | - |
| Singeneic model |  |  |  |  |  |  |
| #11 | yes | - | yes (<0.1) | no | no | no |
| #12 | yes | - | yes (0.83) | no | no | no |
| #13 | yes | - | yes (4.45) | no | no | no |
| #14 | yes | - | yes (2.21) | no | no | no |
| #15 | no (78) | - | yes (<0.1) | no | no | no |
| #16 | yes | - | yes (2.6) | no | no | yes |
| #17 | death (85) | - | - | - | - | - |
| #18 | yes | - | yes (1.64) | no | no | no |

* (day of loss); #(Volume, cm3); -: not pertinent
